# Rational Modification of Estrogen Receptor by Combination of Computational and Experimental Analysis

**DOI:** 10.1371/journal.pone.0102658

**Published:** 2014-07-30

**Authors:** Valentina Elisabetta Viviana Ferrero, Mattia Pedotti, Alessandro Chiadò, Luca Simonelli, Luigi Calzolai, Luca Varani, Teresa Lettieri

**Affiliations:** 1 European Commission - Directorate General - Joint Research Centre, Institute for Environment and Sustainability, Ispra, Varese, Italy; 2 Institute for Research in Biomedicine, Bellinzona, Switzerland; 3 European Commission - Directorate General - Joint Research Centre, Institute for Health and Consumer Protection, Ispra, Varese, Italy; Bioinformatics Institute, Singapore

## Abstract

In this manuscript, we modulate the binding properties of estrogen receptor protein by rationally modifying the amino acid composition of its ligand binding domain. By combining sequence alignment and structural analysis of known estrogen receptor-ligand complexes with computational analysis, we were able to predict estrogen receptor mutants with altered binding properties. These predictions were experimentally confirmed by producing single point variants with up to an order of magnitude increased binding affinity towards some estrogen disrupting chemicals and reaching an half maximal inhibitory concentration (IC_50_) value of 2 nM for the 17α-ethinylestradiol ligand. Due to increased affinity and stability, utilizing such mutated estrogen receptor instead of the wild type as bio-recognition element would be beneficial in an assay or biosensor.

## Introduction

The estrogen receptor protein (ER) is a member of the superfamily of nuclear receptors [1]–[3] whose natural ligand is the hormone 17β-estradiol. Estrogen receptors are present in all vertebrates, highlighting the importance of the ER signal pathway. Binding of 17β-estradiol to ER activates a signaling pathway that regulates several key biological processes such as reproduction, embryonic development [4] and homeostasis [5]–. There are two distinct estrogen receptor genes, resulting in two subtypes of estrogen receptors (ER_α_ and ER_β_) that differ in tissue distribution and ligand preference [8]. In addition to the classical ER ligand inducible transcription activity, there are mounting evidence that ER can act as extra-nuclear activator, independent of gene expression and protein synthesis [9]. These activities are linked to the ERs residing in, or near, the plasma membrane and seem to be involved in breast cancer development and progression[10].

ER is composed by three structural domains: a modulating domain with ligand-independent transactivation function, a DNA-binding domain (DBD) and a ligand binding domain (LBD) [11]–[13]. The amino acid sequences of the LBD of estrogen receptors from several species are available and they indicate that the core of this domain is highly conserved from mammals to fish [14], [15].

Even if sequence homology in the ligand binding domain is high, several studies indicate that estrogenic compounds may have different affinities for ER subtypes [16] and for different organisms [17]–[19].

Besides the natural hormone ligands, a large variety of chemical compounds (collectively referred to as endocrine disrupting chemicals, EDCs) can bind to ERs [20]. Many EDCs have been shown to be toxic for animals and humans due to their ability to interfere with the normal function of ER, leading to many adverse effects such as reproductive problems, hormonal and immune system malfunctions, several types of cancer [21]–[23] and feminization in some fish and amphibians [24]–[31].

Thousands of EDCs, belonging to various chemical classes such as drugs, pesticides, byproducts of plastic and healthcare industries, are commonly present in the environment as a result of industrial, agricultural and household waste [32]. EDCs may also arise from the degradation pathway of otherwise harmless compounds. They are of particular concern due to their wide environmental dispersion and to their tendency to bio-accumulate [33]. It is not possible to classify a compound as an EDC, and thus potentially dangerous, based on its chemical properties alone; instead, the ability of such compound to bind to ER and alter its function should be investigated. Monitoring the presence of a vast number of different EDCs in soil and water and studying their biochemical effect on ER is considered one of the key current challenges for ensuring healthy ecosystems in both developed and in-development countries.

For these reasons, analytical methods that exploit ER as a bio-recognition element to detect the presence of EDCs are particularly attractive: if a chemical binds to ER in the assay, then it means that it can potentially interfere with the hormone signaling pathways and thus be toxic.

X-ray structures have shown that the human ER binds ligands in a highly hydrophobic pocket that can accommodate EDCs of different sizes and chemical properties [34]–[36]. Due to the high sequence identity, it is likely that the general conformation of the ER ligand binding site is conserved; however, local structural differences and a certain degree of conformational flexibility have to be present to account for the different properties and affinities of EDC compounds. These differences may, at least in principle, be exploited for the rational design of modified receptors capable of recognizing classes of EDCs with different affinity and selectivity.

In this manuscript, we aimed to modulate the binding properties of the estrogen receptor protein by rationally modifying the amino acid composition of the ligand binding domain. By combining sequence alignment and structural analysis of known ER-ligand complexes with computational analysis, we were able to predict single point variants of the estrogen receptor ligand binding domain (ER_α_^LBD^) with altered binding properties with respect to the wild type ER ligand binding domain (wt-ER_α_^LBD^). These predictions were experimentally confirmed by producing and characterizing the most relevant recombinant ER_α_^LBD^ variants. In particular, we were able to generate a single point ER mutant with a 6-fold increased binding affinity towards some EDCs (bisphenolic compounds), reaching i.e. an IC_50_ value of 2 nM for 17α-ethinylestradiol ligand. 17α-Ethinylestradiol is an orally bio-active hormone and one of the most commonly used medications, identified as an emerging aquatic pollutant due to its bio-accumulation in surface waters [37], [38]. Due to the increased affinity of one of our ER variants for this and other compounds, utilizing such mutated ER instead of the wt-ER_α_^LBD^ as bio-recognition element in an assay or biosensor would result in increased sensitivity.

## Results

### Sequence and structural analysis of Estrogen Receptors

**Figure 1:**
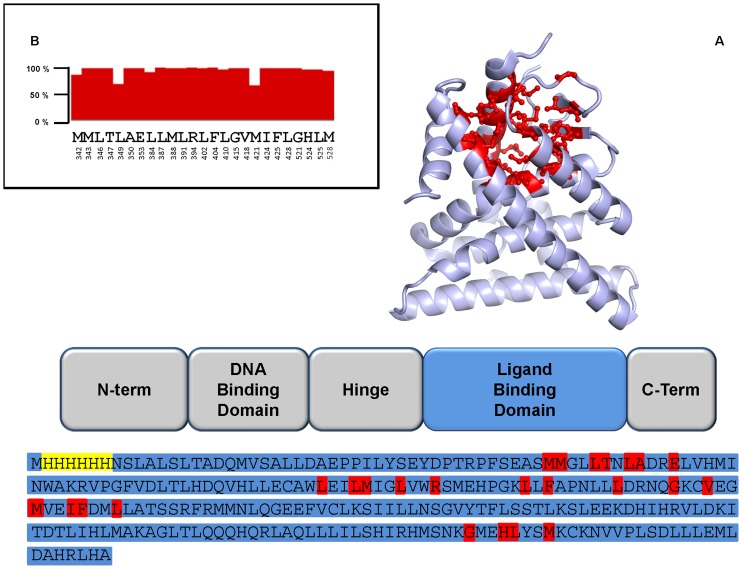
Schematic representation of the structural domains of ER protein. A) A cartoon representation of the three-dimensional structure of the ligand binding domain is shown, as well as its sequence. Residues belonging to the ligand binding pocket are shown in red in both the structure and sequence. Residues highlighted in yellow belong to the histidine tag, residues in light blue encompass the ligand binding domain. B) Degree of conservation for residues of the ligand binding pocket among the analyzed ER sequences. Full bars correspond to 100% conservation.

The full-length Estrogen Receptor α (ER_α_) is a protein of approximately 65 kDa formed by several independent structural domains (Figure 1A). The so-called Ligand Binding Domain (LBD, approximately 25 kDa) is necessary and sufficient to bind either the natural ligand (17β-estradiol) or EDCs.

Comparison of the amino acid sequence of several species shows that the LBD (corresponding to the polypeptide fragment 303-547) is highly conserved. The polypeptide fragment 303-547 of the human ER_α_ has been thus cloned (with the addition of a His_6_ N-terminal sequence for affinity purification) into the expression vector pET21a(+) to produce the recombinant protein used in these studies: ER_α_ (303-547), or in short ER_α_^LBD^.

X-ray crystallography structures have shown the region of the human ER_α_^LBD^ responsible for EDCs binding [34]–[36]. EDCs bind to a highly hydrophobic pocket that can accommodate compounds with different sizes and chemical properties. The residues forming the ligand binding pocket are almost universally conserved but some intriguing differences are nonetheless present.

As a first step we defined an ER_α_^LBD^ residue as belonging to the binding pocket if any of its atoms are within 6 Å of the ligand in any of the x-ray structures of human ER ligand binding domain bound to different EDCs of various sizes. The 6 Å distance cut-off is rather large in order to obtain a conservative description of the binding pocket. This identifies a set of 25 non-contiguous amino acids (human ER_α_ numbering), shown in Figure 1B.

We then compared the sequence alignment of these residues in more than 200 ER proteins from different species. Most residues have 100% conservation with a few exceptions noted below (Figure 1B). Position 349 is predominantly occupied by a methionine (M), but it can also be leucine (L) as in the case of the human protein or, more rarely, valine (V). All three possible variants in position 349 (M, L, V) have aliphatic side chains with similar size and properties. Computational simulations suggest that these mutations should not have a great impact on the binding pocket.

Residue 421 is usually a methionine (M) but it is replaced by isoleucine (I) or leucine (L) in several species, a rather conservative mutation once again. In 22 of the analyzed ER sequences, however, residue 421 is a phenylalanine (F), which is still hydrophobic but has properties not shared by the other amino acids. First of all, F421 introduces an aromatic residue in the binding pocket and it may increase the affinity of the receptor for aromatic compounds such as, for instance, bisphenols. In second order, introducing the large phenylalanine side chain may reduce the size of the ligand binding pocket. The F421-ER_α_^LBD^, thus, may be unable to bind large EDCs due to steric clashes with the phenylalanine aromatic ring. Alternatively, structural changes in the binding pocket may be required to accommodate large EDCs.

Less frequent and conservative amino acid substitutions are also present at position 350, 351, 522, and 525.

### Computational design of mutant ER receptors

To rationally design ER receptors with altered binding properties towards different classes of EDCs, we predicted the structural effects of introducing single point mutations in the ER_α_ binding pocket. The first candidates for virtual mutations analysis were those amino acids that exist as natural variants in different species. This strategy should increase the probability that the resulting mutated protein is functional and properly folded. To this end, the most interesting starting point seemed to be position 421, for which there are existing natural variants carrying either M, L, I, or F amino acids, which provide opportunities for both conservative (M421L, M421I) and non-conservative (M421F) mutations. We used computational approaches to predict: the structure of each ER mutant of interest (M421F, M421I, M421L); the structure of the complexes between each mutant and either the natural 17β-estradiol ligand or some selected EDCs.

To predict the structure of the unbound, mutated ER_α_^LBD^ we either i) replaced the side chain of the interested residue and used rotamer libraries to define its new orientation or ii) predicted the whole protein structure by homology modeling. No significant structural differences were found between wt and mutated ER when unbound. This is not surprising, since even the largest side-chain (F421) can be accommodated in the existing binding pocket if this is not occupied by ligands.

**Figure 2:**
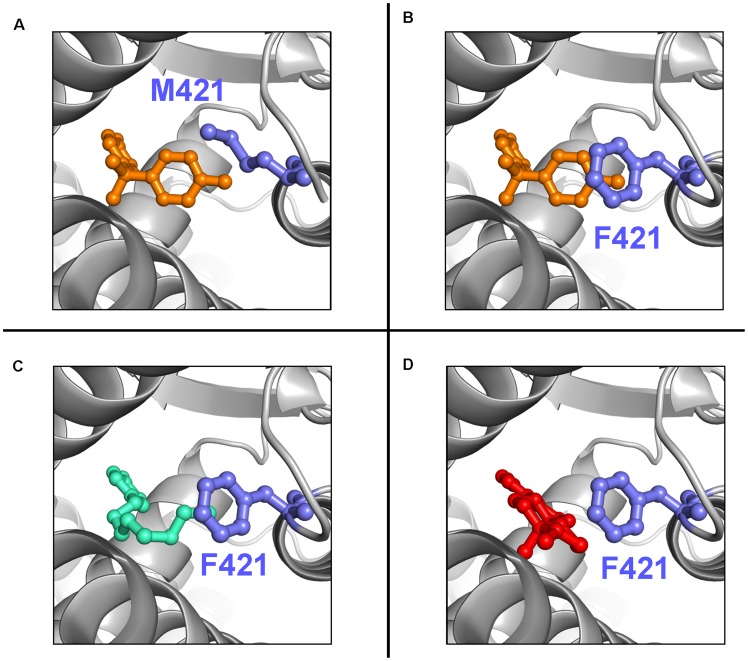
Computational docking models of ligands in the ER binding pocket. The protein backbone is shown as grey cartoon; ligands and side chain of residue 421 are shown as sticks; hydrogen atoms are not shown for clarity. M421F-ER_α_^LBD^ mutant bound to bisphenol-A (orange in B), 4-nonylphenol (green in C) and 17β-estradiol (red in D). In contrast to the wt-ER_α_^LBD^ (shown in A with bisphenol A), the aromatic ring of the F421 mutated ER may increase the affinity for the ligands either by direct stacking interactions or by increasing the aromatic content of the ligand binding pocket; no steric clashes are created by the incorporation of this larger side chain.

We then moved to predict the three-dimensional structure of the complex between wild type and mutated ER and selected EDC ligands. As a first test to assess the accuracy of our docking approach, we predicted the structures of 17β-estradiol and bisphenol-A with human wt-ER (Figure 2A). X-ray structures of each of these are available and the computational results were in very good agreement with the experimental data, supporting the idea that computational predictions can be used to assess the impact of the mutations. We then proceeded to predict the structure of the above mentioned compounds in complex with the ER mutants. We used two different docking algorithms with either rigid or flexible docking options. In the latter case the side-chains and backbone of the protein are allowed to move, trying to accommodate local structural rearrangements upon EDC binding.

It should be pointed out, however, that large changes in the ER structure cannot be correctly simulated and would not be detected by this approach. No significant differences amongst the various procedures were noted.

### Ligand binding to M421F-ER_α_^LBD^

We suspected that introduction of an aromatic ring in the binding pocket (M421F mutant) could increase the affinity of ER for aromatic compounds. Indeed, computational docking predictions suggest that bisphenol-A bound to M421F-ER_α_^LBD^ changes in comparison to its complex with wt-ER_α_^LBD^, bringing one of its aromatic rings closer to the ring of F421 (Figure 2B), something that cannot be achieved with wt-ER_α_^LBD^ (Figure 2A) This may result in a higher affinity of bisphenol-A for M421F-ER_α_^LBD^, either due to the formation of direct aromatic stacking interactions or as a consequence of the increased aromatic character of the mutated binding pocket.

Phenolic compounds linked to bulkier aliphatic side chains (e.g. 4-nonylphenol) are somehow different: although they can fit in the mutated ligand binding pocket just like wt-ER_α_^LBD^, their larger size does not allow their aromatic ring to get closer to M421F as it happens to bisphenol-A (Figure 2C). We wouldn't expect, therefore, an increased affinity of M421F-ER_α_^LBD^ for these compounds.

The natural ER ligand, 17β-Estradiol, has an aromatic ring that may be favorably affected by the M421F substitution in a similar way to bisphenols (Figure 2D). However, the aromatic ring is far away from M421 in the wt-ER_α_^LBD^ experimental structure; if F421 would maintain the same conformation, it would be unable to achieve direct aromatic stacking interactions with 17β-Estradiol. On the other hand, if 17β-estradiol would bind to F421 in a position different from the one it has in wt-ER_α_^LBD^, then the M421F-ER_α_^LBD^ could have increased affinity for 17β-estradiol due to favorable interactions between the aromatic rings. Computational docking of 17β-estradiol in complex with M421F-ER_α_^LBD^ shows a binding conformation comparable to that of wt-ER_α_^LBD^ in the models considered more energetically favorable by the algorithm (Figure 2D). Models deemed to be less stable, however, position the aromatic ring of 17β-estradiol in proximity of the ring of F421, which may result in increased binding affinity. Since computational algorithms are notoriously unreliable at correctly identifying energetically favored ligand positions within the same binding region, it can be suggested that 17β-estradiol can fit in the mutated binding pocket with its aromatic ring close to F421. Whether this conformation is preferred in vivo remains to be seen.

Finally, computational docking of tamoxifen (a commercial drug that binds to ER) shows no appreciable differences between wt-ER_α_^LBD^ and M421F-ER_α_^LBD^. Although tamoxifen is much larger than the natural 17β-estradiol ligand, x-rays structure has shown it to only partially occupy the ligand binding pocket [34], which can thus probably be restricted without affecting the ligand.

### Production and characterization of ER mutants

Structural and computational analysis of the wt-ER_α_^LBD^ binding pocket suggested that mutations in position 421 could have a significant influence on the affinities of different EDCs towards the ER. In order to verify this hypothesis by experimental measures of binding affinity we generated three ER mutants: M421F-ER_α_^LBD^, M421I-ER_α_^LBD^, and M421L-ER_α_^LBD^.

**Figure 3:**
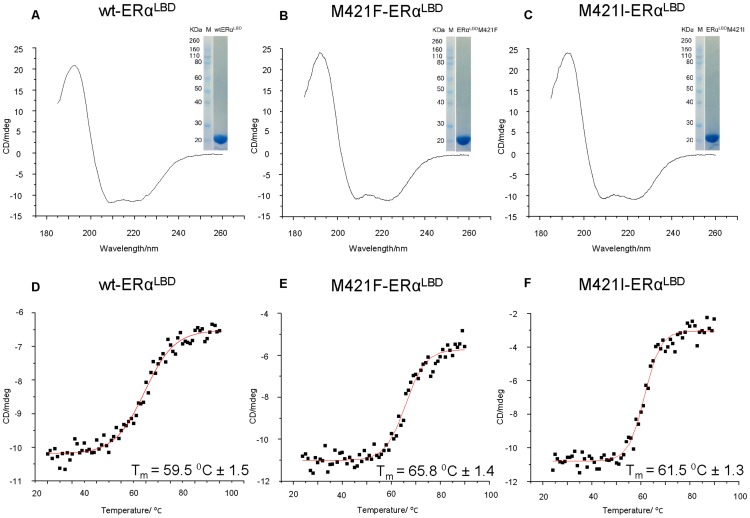
Circular dichroism and SDS-PAGE of recombinant ER proteins. CD spectra show typical α-helical character and a single band at the expected molecular weight is visible (insets). (A) Far-UV CD and SDS-PAGE of wt-ER_α_^LBD^ (B) Far-UV CD and SDS-PAGE of M421F-ER_α_^LBD^ (C) Far-UV CD and SDS-PAGE of M421I-ER_α_^LBD^ (D) – (F): thermal unfolding data of the three proteins. Intensity of peak at 222 nm plotted as a function of increased temperature. Melting temperature (T_m_) reported.

High yield of proteins, ranging from 25 to 30 mg/L of liquid culture, were obtained. Following a single purification step by affinity chromatography pure proteins samples were obtained, as shown in the SDS-PAGE of the various ER mutants (inset in Figures 3A, B and C).

CD spectroscopy confirms that the mutants are properly folded (Figures 3B and 3C) and comparable to wt-ER_α_^LBD^ (Figure 3A). The percentage of secondary structure elements were calculated using standard algorhythms provided by the online DichroWeb service [39]. The calculations showed the presence of 54% α-helical secondary structure for all the wild-type and the mutants which reflects well the secondary structure elements derived from the available three dimensional structure of wt-ER_α_^LBD^ (PDB: 1ERE).

The thermal stability of the different ERs was assessed by following the protein thermal unfolding between 25 and 90°C with CD spectroscopy (Figures 3D, 3E and 3F). The unfolding process is not reversible: by cooling back the samples to 25°C only around 50% of the original secondary structure of the protein is recovered (data not shown). By fitting the experimental data with a Boltzmann-type equation it was possible to calculate melting temperature (T_m_) values of 59.5±1.5°C, 65.8±1.4°C, and 61.5±1.3°C for the wt-ER_α_^LBD^, M421F-ER_α_^LBD^, and M421I-ER_α_^LBD^ proteins, respectively.

The M421L-ER_α_^LBD^ mutant had the same characteristics of purity, secondary structure and melting temperature of M421I-ER_α_^LBD^ (data not shown).

The melting temperature of the wt-ER_α_^LBD^ receptor is about 20°C higher than that previously reported for the full-length ER_α_ protein [40], [41]. The isolated ligand binding domain, in other words, appears more stable than the full length ER protein. It is also interesting to note that it is reported that the CD signal decreases sharply as the temperature increases from 20 to 30°C and then increases gradually from 30 to 70°C. We, instead, observe a single transition sigmoidal trend. Our results also indicate that the M421F-ER_α_^LBD^ mutant is significantly more stable than the wt-ER_α_^LBD^.

### Competitive binding assay

**Figure 4:**
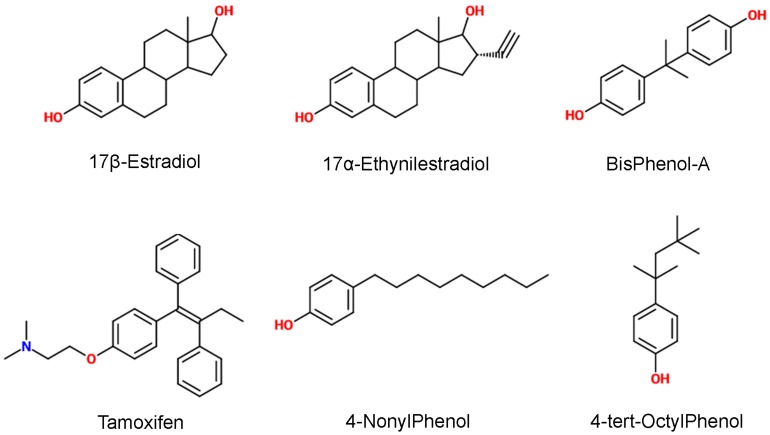
Chemical structures of tested compounds.

The PolarScreen assay was used to test the ligand binding affinity of wt-ER_α_^LBD^ and its mutants towards different classes of estrogen disrupting compounds. The tested chemical compounds (shown in Figure 4) were: the natural ligand 17β-estradiol; its close analog 17α-ethynilestradiol (chosen since it is an emerging pollutant [42]); tamoxifen, a commercial drug antagonist often detected in surface waters [43]; and three other compounds with known estrogen disrupting activity and significant presence in the environment, bisphenol-A, 4-nonylphenol and 4-tert-octylphenol [44]. The latter three have micromolar binding affinity whereas the first three have nanomolar affinity for wild type ER [45].

As a first test we compared the binding affinity of our wt-ER_α_^LBD^ protein with that of the full length ER supplied with the kit. The binding affinities for the 17β-Estradiol ligand were measured as 12±2 nM and 16±4 nM for the full length and wt-ER_α_^LBD^ respectively, indicating that the isolated ligand binding domain is just as active as the full protein in terms of ligand binding.

**Figure 5:**
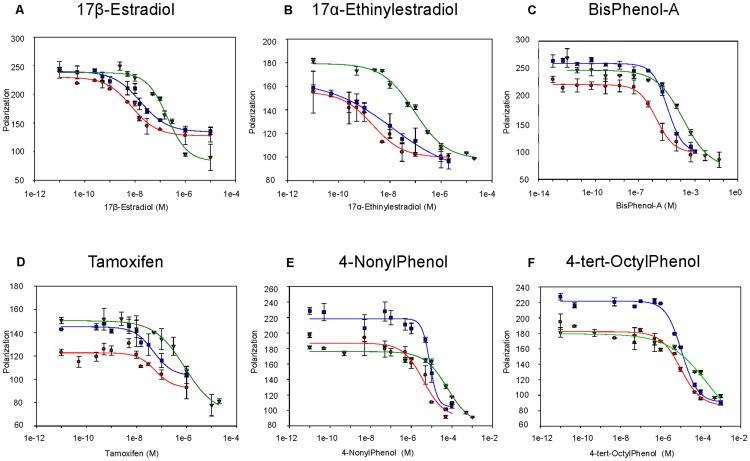
Competitive binding assay. Competitive binding assay on wt-ER_α_^LBD^ (blue squares), M421F-ER_α_^LBD^ (red circles) and M421I-ER_α_^LBD^ (green triangles) with six different compounds: 17β-estradiol (panel A), 17α-ethinylestradiol (panel B), bisphenol-A (panel C), tamoxifen (panel D), 4-nonylphenol (panel E) and 4-tert-octylphenol (panel F).

After this control, we measured the binding affinity of the above mentioned compounds for the recombinant receptors (wt and mutants; results are shown in Figure 5 and Table 1).

**Table 1:** IC_50_ values and standard errors resulting from the competitive binding assay performed in four replicates with wt-ER_α_^LBD^ (column 2), M421F- ER_α_^LBD^ (column 3) and M421I- ER_α_^LBD^ (column 4) and selected compounds (ligands, column 1): 17β-estradiol, 17α-ethinylestradiol, bisphenol-A, tamoxifen, 4-nonylphenol and 4-tert-octylphenol.

	IC_50_		
LIGANDS	wt-ER_α_^LBD^	M421F-ER_α_^LBD^	M421I-ER_α_^LBD^
17β-Estradiol	16±4 nM	7±2 nM	191±50 nM
17α-Ethinylestradiol	12±6 nM	2.1±0.9 nM	90±21 nM
Bisphenol-A	11±2.5 µM	1.9±0.5 µM	100±9 µM
Tamoxifen	47±14 nM	55±40 nM	830±300 nM
4-Nonylphenol	8±1 µM	4±2 µM	60±30 µM
4-Tert-octylphenol	11.4±1.5 µM	10±4 µM	200±60 µM

The M421F-ER_α_^LBD^ shows lower IC_50_, compared to wt-ER_α_^LBD^, for four compounds (17β-estradiol, 17α-ethinylestradiol, bisphenol-A and 4-nonylphenol) out of six.

The M421I-ER_α_^LBD^ has higher IC_50_, compared to wt-ER_α_^LBD^, for all the tested compounds.

Each compound was tested in a range of 12 concentrations with the ER receptors (Figure 5). The compounds concentrations (X axis) were plotted in a logarithmic scale against the Polarization values (Y axis). The plot of compounds concentrations against Polarization were fitted with a one site competition four parameters logistic curve (see Materials and Methods for further details).

The half maximal inhibitory concentration (IC_50_) values of the wt-ER_α_^LBD^ towards the selected compounds agreed with literature data [18], [46]. Differences in protein preparation and assay conditions may contribute to the small variability seen among literature and present data.

The IC_50_ of the mutant M421F-ER_α_^LBD^ (red circles in Figure 5) with most compounds was different from the wt-ER_α_^LBD^ (blue squares in Figure 5). In particular, IC_50_ values indicate that 17α-ethinylestradiol and bisphenol-A bind approximately 6 times more strongly to M421F-ER_α_^LBD^ than to wt-ER_α_^LBD^ (Figure 5 and Table 1). 17β-Estradiol and 4-nonylphenol bind twice as strong to M421F-ER_α_^LBD^, as well. No differences were observed for tamoxifen and 4-tert-octylphenol whose IC_50_ values are not significantly different between M421F-ER_α_^LBD^ and wt-ER_α_^LBD^ (Figure 5 panels D and F).

Comparing the IC_50_ values of the mutant M421I-ER_α_^LBD^ (green triangles in Figure 5) with the wt-ER_α_^LBD^ (blue squares in Figure 5) we observe, instead, that in all cases the mutation negatively affects the binding affinity, with IC_50_ values between 5 and 20 times higher. The M421L-ER_α_^LBD^ gave the same results as M421I-ER_α_^LBD^ and for this reason was not further considered. Repeated measurements were performed over a 9 months period with proteins stored at −20°C; the low standard errors suggest that the proteins are stable, retaining not only their tertiary structure but also their binding ability for at least 9 months. This is an encouraging observation about the possibility to use these receptors as bio-recognition elements in an assay to detect EDCs.

## Discussion

The possibility of rationally modify ER receptors with improved affinities towards different classes of endocrine disrupting chemicals would allow both the development of analytical tools for the rapid screening and detection of EDCs and a better understanding of the their structure-activity relationship. To this end the combination of structural analysis and computational modeling allowed the design of mutants predicted to have different affinities towards different EDC classes. Indeed, we obtained mutated ERs with 2 to 6 fold increased binding affinity for 17β-estradiol, 17α-ethinylestradiol, bis-phenol-A and 4-nonylphenol.

Computational docking simulations suggested that the mutant M421F-ER_α_^LBD^ would have improved binding affinity for at least some chemical compounds, as it was later verified by experimental results. Generally speaking, docking results cannot be taken at face value, especially when comparing binding energies. However, they do provide useful hints about what is feasible and what not, thus guiding the rational design of mutants. In the case of M421F-ER_α_^LBD^, simulations indicated that introduction of an aromatic side chain could favor binding of aromatic compounds, either through direct stacking interactions between the aromatic rings of chemicals and protein side chains or simply by increasing the overall aromatic content of the ligand binding pocket. It is also possible that the mutation stabilizes transient intermediates that facilitate passage of the organic compound from the media to the buried ligand binding pocket, although we have no direct evidence for this.

In contrast to the M421F mutation, the M421I restricts the binding pocket (Isoleucine has a bulkier side chain than Methionine) without increasing the aromatic content. This reduces the binding affinities of all the tested compounds by 8 to 20 folds.

The *in-silico* designed mutants, M421F-ER_α_^LBD^ and M421I-ER_α_^LBD^, were properly folded and active in solution, as shown by their CD spectra and binding properties. These findings highlight the advantages of sequence analysis and the use of mutations that are rare but yet present as natural variants in some species. This approach highly increases the possibility that the resulting mutants are properly folded and active, something that is not always guaranteed when mutants are generated through randomization technologies or other strategies. As an added bonus, the isolated ligand binding domain that we used proved to be more stable than the full-length ER protein used in other published or commercial assays. Introduction of the rational mutation M421F further increased protein stability, both in terms of increased resistance to thermal denaturation (as proved by the increase of the melting temperature from 60 to 66°C) and of ligand binding activity after prolonged storage.

The M421F-ER_α_^LBD^ with increased stability and higher binding affinities towards selected EDCs could be used as the capture agent in affinity chromatography. For example, our recombinant ER_α_^LBD^ receptor was recently used for the fast detection of estrogens in dietary supplements [47]. In addition, the availability of ER proteins with variable binding affinities (i.e. wild type, M421F and M421I mutants) could be used in array format assays to not only detect EDCs but also to discriminate amongst different EDC classes. Moreover, this kind of receptors can be used as bio-recognition element for label-free detection by means of highly sensible techniques, such as those based on SPR (Surface Plasmon Resonance), QCM (Quartz Crystal Microbalance) or MC (Micro Cantilever) [48]. Such detection systems could be then applied for the EDC screening in complex matrices such as food, aquaculture, fresh and seawater as well as for screening of chemicals with potential EDC activity.

Our results indicate, as a proof of concept, that the combination of structural and sequence analysis with computational simulations, allow the successful rational design of ER mutants with desired binding properties. We think that the workflow illustrated in this manuscript could be successfully applied to the rational design of other ER mutants or to the modification of other ligand binding proteins.

## Materials and Methods

### Materials

The wild-type estrogen receptor α ligand binding domain encoding gene was synthesized and cloned into pET21a(+) by Genscript (Piscataway, NJ, USA). The QuikChange Site-Directed-Mutagenesis Kit was purchased by Qiagen (Hilden, Germany), while primers and sequencing were done by Primm (Milan, Italy).

The Bacto Yeast Extract and Bacto Tryptone for the Luria Bertani (LB) medium were from BD Biosciences (Franklin Lakes, NJ, USA). Rosetta 2(DE3)pLysS Competent Cells come from Merck.

Isopropyl β-D-1-thiogalactopyranoside (IPTG), Tris-HCl, NaCl, β-mercaptoethanol (β-Me), 3-(1-pyridinio)-1-propanesulfonate (NDSB) and the test compounds 17β-estradiol, 17α-ethynilestradiol, bisphenol-A, tamoxifen, 4-nonylphenol and 4-tert-octylphenol were supplied by Sigma Aldrich.

Nickel HiLoad Column and the AKTA System purifier are from GE Healthcare. Life Technologies supplied the PolarScreen Estrogen Receptor-α Competitor Green Assay.

### Alignment and Computational analysis

Estrogen receptor protein sequences from several species were retrieved through the Expasy protein server database (http://www.expasy.org/). Sequence alignment was performed according to standard procedures using the Jalview program suite and included algorithms (http://www.jalview.org/). Duplicate and incomplete sequences were manually removed from the analysis.

### Structural predictions of mutated ER

The X-ray structure of a human estrogen receptor complexed with bisphenol-A (PDB: 3UU7) was used as a starting PDB file. Mutants model structures were predicted by Pymol [49] using the wild type as a template structure and changing the side chain conformation according to rotamer libraries. Homology modeling was performed with the i-Tasser web server [50], [51]. The PDB files of wild type and mutant model structures were prepared for docking using the dockprep tool in UCSF Chimera package [52], while bisphenol-A was prepared using MarvinSketch (Marvin 5.9.0, http://www.chemaxon.com/) and OpenBabel [53]. Computational docking was performed by means of SwissDock server (www.swissdock.ch) based on the docking software EADock DSS [54], with a user-defined box (15Åx 15Åx 15Å) centered on the receptor binding site of the LDB. Briefly, a tree-based Dihedral Space Sampling (DSS) algorithm generates 15000 binding modes that are subsequently minimized (100 steps of steepest descent algorithm and 250 steps of adopted basis Newton Raphson algorithm). Redundant binding modes and binding modes making little or detrimental interactions with the protein are removed. Simultaneously, the CHARMM [55] energies of the remaining binding modes are estimated on a grid. Then, binding modes with the most favorable energies are ranked, taking account of the solvent effect using the FACTS implicit solvation model [56], and clustered by root mean square deviation (RMSD) with a distance cutoff of 2 Å. Finally, the most favorable clusters are ranked by an estimated ΔG of binding. Selected calculations were repeated with the docking program Autodock 4.2 [57] with default options and similar search boundaries as above. Docking was performed either with rigid or flexible body. The latter option allows for limited movement of protein side chain and backbone. No significant differences were noted between the various algorithms. Subsequent molecular graphics and analyses of docking results were performed with the UCSF Chimera package [52], or with Pymol [49]. 3D structures were analyzed with the program Pymol.

In an attempt to cover the different conformations allowed to the F421 mutant, we generated four different models in which the aromatic side-chain occupies four different positions, defined by either rotamer libraries (using Pymol) or de novo structural predictions (using the iTASSER server). We then used each of these models independently in subsequent docking simulations. As a validation step for our approach we docked EDC/ER complexes with known experimental structure. Evaluation of the spatial distance of the ligand position between experimental and predicted structure (RMSD) showed that solutions nearly identical to the experimental ones were selected by the computational algorithm as most energetically favorable. This suggests that the approach can give reliable information also for complexes with unknown experimental structure.

### Generation of mutated plasmids

In order to selectively mutate the methionine 421 of the wt-ER_α_^LBD^ into phenilalanine, leucine or isoleucine, the plasmid containing the wild type gene was used as starting material.

To generate the M421F, M421I and M421L variants two mutagenic primers (forward and reverse) were designed for each single mutation according to indications on the QuikChange Site-Directed-Mutagenesis Kit (Qiagen) with a length between 25 and 45 base pairs, T_m_≥78°C.

Forward Primer M421F: 5′- AGGCAAATGCGTCGAGGGTTTTGTGGAAATTTTTGACATGC-3′

Reverse Primer M421F: 5′- GCATGTCAAAAATTTCCACAAAACCCTCGACGCATTTGCCT -3′

Forward Primer M421I: 5′-GCAAATGCGTCGAGGGTATTGTGGAAATTTTTGACATG-3′

Reverse Primer M421I: 5′-CATGTCAAAAATTTCCACAATACCCTCGACGCATTTGC-3′

Forward Primer M421L: 5′-ATCAAGGCAAATGCGTCGAGGGTCTGGTGGAAATTT-3′

Reverse Primer M421L: 5′-AAATTTCCACCAGACCCTCGACGCATTTGCCTTGAT-3′

Each reaction volume contained the reaction buffer, plasmid pET21a(+)-wt-ER_α_^LBD^, the two primers, dNTP mix, and water. The PFU-Turbo polymerase (QuikChange Site-Directed-Mutagenesis Kit, Qiagen) was added and the PCR reaction was performed in a PCR Thermocycler (Stratagene). After the PCR, the DpnI enzyme (Qiagen) was added to digest the parent DNA and then the mixture was used to transform XL1Blue competents cells (Qiagen). The extracted plasmids from the positive colonies were sequenced (Primm).

### Protein expression and purification

The pET21a(+) plasmid containing the ER gene was used to transform Rosetta 2(DE3)pLysS Competent Cells (Merck), plated on ampicillin selective LB-agar and incubated over night at 37°C. Different *E.coli* strains, such as One Shot BL21 Star (DE3), Rosetta 2(DE3) and Rosetta 2(DE3) pLysS were tested and the latter ones were chosen because they showed the best yield in terms of purity and amount (mg/l culture) of the expressed protein due to the pLysS plasmid that expresses T7 lysozyme [58] .

The cells are then grown in liquid culture (LB with 10% sucrose) at 37°C until an OD_600_ of 0.7 is reached, then IPTG 0.5 mM and 3% ethanol are added. The protocol was modified starting from the one of Ahrens et al. [59]. Afterwards cells are grown at 18°C for 18 hours; washed once in buffer A (50 mM Tris-HCl, 50 mM NaCl, 10 mM β-mercaptoethanol, 10 mM ((3-(1-pyridinio)-1-propanesulfonate), 1 tablet of complete protease inhibitor, and 20 mM imidazole), then sonicated and centrifuged at 18000 rpm for 1 h at 4°C. The supernatant is then loaded on a 5 mL Nickel HiLoad Column (GE Healthcare) connected to an AKTA system purifier while the pellet was resuspended in Tris-HCl 50 mM pH 7.5 and Urea 6 M (5 mL per liter of culture) to extract additional proteins from the cell debris and left reacting for 5 minutes, then centrifuged at 18000 rpm for 1 h at 4°C.

The Nickel HiLoad column is equilibrated with washing buffer 1X (50 mM Tris-HCl pH 7.5, 500 mM NaCl, 20 mM imidazole, 10 mM β-mercaptoethanol) prior to supernatant loading. After washing out the unbound proteins with 4 column volumes (20 mL), the elution of the His-tagged protein is performed by a linear gradient from 0 to 100% of elution buffer (50 mM Tris-HCl pH 7.5, 300 mM NaCl, 500 mM imidazole, 10 mM β-mercaptoethanol). The fractions that absorb at 280 nm are run on a SDS-PAGE gel to visualize the presence of the protein.

The purified fractions containing the protein are dialyzed in storage buffer (Tris-HCl 20 mM pH 7.4 and NaCl 150 mM) to remove the imidazole and to lower the salt concentration. The purified protein was then stored at −20°C.

### Protein characterization with Circular Dichroism (CD) spectroscopy

Circular dichroism spectra were recorded with a J750 Circular dichroism spectrometer from Jasco interfaced with a Peltier temperature control unit with 1 mm path length cuvette. Analysis of the far-UV region (185–260 nm) was used to investigate the secondary structure and the folding of the protein. CD spectra were analyzed with the DichroWeb [39] online software, by means of the CDSSTR program [60]. Thermal denaturation experiments were performed by monitoring the circular dichroism at 222 nm while changing the temperature from 25 to 90°C (1°C/min) and backward. Reference spectra were collected for the buffers in which proteins were dissolved. Denaturation curves were analyzed assuming a two-state unfolding model and melting temperature (T_m_) was calculated for every mutant [61]. All the statistical analysis and fittings were performed with Sigma Plot 12.3.

### Competitive binding assay

To test the binding affinity of the recombinant receptor for EDCs we used the competitive binding assay developed by Life Technologies for ER_α_, the PolarScreen Estrogen Receptor-α Competitor Green Assay. Although radioactive binding assays are widely used for ER, fluorescence assays are just as reliable but do not require radioactive safety procedures making them more convenient.

The full length ER_α_ provided with the kit was replaced by our recombinant ER_α_^LBD^. In short, wt or mutant ER_α_^LBD^ are added to the Fluormone and incubated at 4°C for 45 minutes to form the receptor-Fluormone complex. This complex is then mixed and incubated at 25°C for 2 h with the individual EDCs and the intensity of the fluorescence polarization signal (directly proportional to the amount of Fluormone bound to the protein) measured. The addition of competitors displaces the Fluormone from the ER_α_^LBD^ receptor, resulting in a decreased fluorescence signal. The fluorescence polarization anisotropy signal (P) is read using an Infinite 200 Pro multimode plate reader from Tecan and calculated with the following equation: 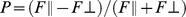



Where:

*F_∥_* =  Fluorescence intensity parallel to the excitation plane,

*F _⊥_*  =  Fluorescence intensity perpendicular to the excitation plane.

The P data (y) were fitted against the competitor concentrations (x) with a typical one site competition three parameters logistic curve:
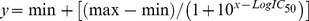



Where:

min and max are respectively the minimum and maximum polarization values, 

*LogIC_50_*  =  Log of the concentration of test compound required to reduce the maximum polarization value by 50%. All the resulting IC_50_ values are obtained by the average of at least four different experiments.

## References

[1] Beato M (1989) Gene regulation by steroid hormones. Cell 56: 335–344. 2644044 10.1016/0092-8674(89)90237-7

[2] Katzenellenbogen BS, Katzenellenbogen JA (2000) Estrogen receptor transcription and transactivation. Estrogen receptor alpha and estrogen receptor beta: regulation by selective estrogen receptor modulators and importance in breast cancer. Breast Cancer Research 2: 335–344. 11250726 10.1186/bcr78 PMC138655

[3] Pettersson K, Gustafsson JA (2001) Role of estrogen receptor beta in estrogen action. Annual Review of Physiology 63: 165–192. 10.1146/annurev.physiol.63.1.165 11181953

[4] Chung AC, Cooney AJ (2003) The varied roles of nuclear receptors during vertebrate embryonic development. Nuclear Receptor Signaling 1: 1–7. 10.1621/nrs.01007 PMC1402219 16604179

[5] Strauss L, Kallio J, Desai N, Pakarinen P, Miettinen T, et al (2009) Increased exposure to estrogens disturbs maturation, steroidogenesis, and cholesterol homeostasis via estrogen receptor a in adult mouse Leydig cells. Endocrinology 150: 2865–2872. 19196801 10.1210/en.2008-1311

[6] Colasanti T, Maselli A, Conti F, Sanchez M, Alessandri C, et al (2012) Autoantibodies to estrogen receptor a interfere with T lymphocyte homeostasis and are associated with disease activity in systemic lupus erythematosus. Arthritis and Rheumatism 64: 778–787. 21968947 10.1002/art.33400

[7] Ribas V, Drew BG, Le JA, Soleymani T, Daraei P, et al (2011) Myeloid-specific estrogen receptor a deficiency impairs metabolic homeostasis and accelerates atherosclerotic lesion development. PNAS 108: 16457–16462. 21900603 10.1073/pnas.1104533108 PMC3182726

[8] Mosselman S, Polman J, Dijkema R (1996) ER beta: identification and characterization of a novel human estrogen receptor. FEBS Letters 392: 49–53. 8769313 10.1016/0014-5793(96)00782-x

[9] Fu X, Simoncini T (2008) Extra-nuclear Signaling of Estrogen Receptors. IUBMB Life 60: 502–510. 18618586 10.1002/iub.80

[10] Pietras RJ, Márquez-Garbán DC (2007) Membrane-associated estrogen receptor signaling pathways in human cancers. Clinical Cancer Research 13: 4672–4676. 17699844 10.1158/1078-0432.CCR-07-1373

[11] Kumar R, Thompson EB (1999) The structure of the nuclear hormone receptors. Steroids 64: 310–319. 10406480 10.1016/s0039-128x(99)00014-8

[12] Shiau AK, Barstad D, Radek JT, Meyers MJ, Nettles KW, et al (2002) Structural characterization of a subtype-selective ligand reveals a novel mode of estrogen receptor antagonism. Nature Structural Biology 9: 359–364. 11953755 10.1038/nsb787

[13] Pike AC, Brzozowski AM, Hubbard RE, Bonn T, Thorsell AG, et al (1999) Structure of the ligand-binding domain of oestrogen receptor beta in the presence of a partial agonist and a full antagonist. EMBO Journal 18: 4608–4618. 10469641 10.1093/emboj/18.17.4608 PMC1171535

[14] Seielstad DA, Carlson KE, Kushner PJ, Greene GL, Katzenellenbogen JA (1995) Analysis of the structural core of the human estrogen receptor ligand binding domain by selective proteolysis/mass spectrometric analysis. Biochemistry 34: 12605–12615. 7548010 10.1021/bi00039a016

[15] Ruff M, Gangloff M, Wurtz JM, Moras D (2000) Estrogen receptor transcription and transactivation: structure-function relationship in DNA- and ligand-binding domains of estrogen receptors. Breast Cancer Research 2: 353–359. 11250728 10.1186/bcr80 PMC138657

[16] Kuiper GG, Lemmen JG, Carlsson B, Corton JC, Safe SH, et al. (1998) Interaction of estrogenic chemicals and phytoestrogens with estrogen receptor beta. Endocrinology 139: 4252–4263. 10.1210/endo.139.10.6216 9751507

[17] Matthews J, Zacharewski TR (2000) Differential binding affinities PCBs, HO-PCBs and Aroclors with recombinant human, rainbow trout (*Onchorhynkiss mykiss*) and reptilian (*Anolis carolinensis*) estrogen receptors using a semi-high throughput competitive binding assay. Toxicological Sciences 53: 326–339. 10696781 10.1093/toxsci/53.2.326

[18] Matthews J, Celius T, Halgren R, Zacharewski T (2000) Differential estrogen receptor binding of estrogenic substances: a species comparison. Journal of Steroid Biochemistry & Molecular Biology 74: 223–234. 11162928 10.1016/s0960-0760(00)00126-6

[19] Le Drean Y, Kern L, Pakdel F, Valotaire Y (1995) Rainbow trout estrogen receptor presents an equal specificity but a differential sensitivity for estrogens than human estrogen receptor. Molecular and Cellular Endocrinology 109: 27–35. 10.1016/0303-7207(95)03482-m 7789614

[20] Sumpter JP (1998) Xenoendocrine disrupters - environmental impacts. Toxicology Letters 102–103: 337–342. 10.1016/s0378-4274(98)00328-2 10022275

[21] Diamanti-Kandarakis E, Bourguignon JP, Giudice LC, Hauser R, Prins GS, et al (2009) Endocrine-disrupting chemicals: an endocrine society scientific statement. Endocrine Reviews 30: 293–342. 19502515 10.1210/er.2009-0002 PMC2726844

[22] Damstra T (2002) Potential effects of certain persistent organic pollutants and endocrine disrupting chemicals on the health of children. Journal of Toxicology and Clinical Toxicology 40: 457–465. 12216998 10.1081/clt-120006748

[23] Watanabe H, Suzuki A, Kobayashi M, Lubahn DB, Handa H, et al (2003) Similarities and differences in uterine gene expression patterns caused by treatment with physiological and non-physiological estrogens. Journal of Molecular Endocrinology 31: 487–497. 14664709 10.1677/jme.0.0310487

[24] Colborn T (2002) Clues from wildlife to create an assay for thyroid system disruption. Environmental Health Perspectives 110: 363–367. 10.1289/ehp.02110s3363 PMC1241184 12060830

[25] Tyler CR, Jobling S, Sumpter JP (1998) Endocrine disruption in wildlife: a critical review of the evidence. Critical Review of Toxicology 28: 319–361. 10.1080/10408449891344236 9711432

[26] Harries JE, Sheahan DA, Jobling S, Matthiessen P, Neall P, et al (1997) Estrogenic activity in five United Kingdom rivers detected by measurement of vitellogenesis in caged male trout. Environmental Toxicolology and Chemistry 16: 534–542.

[27] Noaksson E, Tjärnlund U, Bosveld AT, Balk L (2001) Evidence for endocrine disruption in perch (*Perca fluviatilis*) and roach (*Rutilus rutilus*) in a remote swedish lake in the vicinity of a public refuse dump. Toxicology and Applied Pharmacology 174: 160–176. 11446832 10.1006/taap.2001.9194

[28] Viganò L, Benfenati E, Bottero S, Cevasco A, Monteverde M, et al (2010) Endocrine modulation, inhibition of ovarian development and hepatic alterations in rainbow trout exposed to polluted river water. Environmental Pollution 158: 3675–3683. 20864230 10.1016/j.envpol.2010.07.033

[29] Kidd KA, Blanchfield PJ, Mills KH, Palace VP, Evans RE, et al (2007) Collapse of a fish population after exposure to a synthetic estrogen. PNAS 104: 8897–8901. 17517636 10.1073/pnas.0609568104 PMC1874224

[30] Allen Y, Matthiessen P, Scott AP, Haworth S, Feist S, et al (1999) The extent of oestrogenic contamination in the UK estuarine and marine environments - further surveys of flounder. The Science of the Total Environment 233: 5–20. 10492895 10.1016/s0048-9697(99)00175-8

[31] Hashimoto S, Bessho H, Hara A, Nakamura M, Iguchi TF (2000) K (2000) Elevated serum vitellogenin levels and gonadal abnormalities in wild male flounder (*Pleuronectes yokohamae*) from Tokyo Bay, Japan. Mar Environ Res 49: 37–53. 11444013 10.1016/s0141-1136(99)00047-1

[32] Muller M, Rabenoelina F, Balaguer P, Patureau D, Lemenach K, et al (2008) Chemical and biological analysis of endocrine-disrupting hormones and estrogenic activity in an advanced sewage treatment plant. Environmental Toxicology and Chemistry 27: 1649–1658. 18315391 10.1897/07-519

[33] Liu J, Wang R, Huang B, Lin C, Zhou J, et al (2012) Biological effects and bioaccumulation of steroidal and phenolic endocrine disrupting chemicals in high-back crucian carp exposed to wastewater treatment plant effluents. Environmental Pollution 162: 325–331. 22243881 10.1016/j.envpol.2011.11.036

[34] Shiau AK, Barstad D, Loria PM, Cheng L, Kushner PJ, et al (1998) The structural basis of estrogen receptor/coactivator recognition and the antagonism of this interaction by tamoxifen. Cell 95: 927–937. 9875847 10.1016/s0092-8674(00)81717-1

[35] Tanenbaum DM, Wang Y, Williams SP, Sigler PB (1998) Crystallographic comparison of the estrogen and progesterone receptor's ligand binding domains. Proceedings of the National Academy of Sciences of the United States of America 95: 5998–6003. 9600906 10.1073/pnas.95.11.5998 PMC27574

[36] Kim S, Wu JY, Birzin ET, Frisch K, Chan W, et al (2004) Estrogen receptor ligands. II. Discovery of benzoxathiins as potent, selective estrogen receptor alpha modulators. Journal of Medicinal Chemistry 47: 2171–2175. 15084115 10.1021/jm034243o

[37] Snyder SA, Lei H, EC W (2008) Removal of endocrine disruptors and pharmaceuticals during water treatment. New York: CRC Press 229–259 p.

[38] Lai KM, Scrimshaw MD, Lester JN (2002) Prediction of the bioaccumulation factors and body burden of natural and synthetic estrogens in aquatic organisms in the river systems. The Science of the Total Environonment 289: 159–168. 10.1016/s0048-9697(01)01036-1 12049392

[39] Whitmore A, Wallace BA (2008) Protein secondary structure analyses from circular dichroism spectroscopy: methods and reference databases. Biopolymers 89: 392–400. 17896349 10.1002/bip.20853

[40] Greenfield NJ, Vijayanathan V, Thomas TJ, Gallo MA, Thomas T (2001) Increase in the stability and helical content of estrogen receptor in the presence of the estrogen response element: analysis by circular dichroism spectroscopy. Biochemistry 40: 6646–6652. 11380259 10.1021/bi002846l

[41] Nair SK, Thomas TJ, Greenfield NJ, Chen A, He H, et al (2005) Conformational dynamics of estrogen receptors and as revealed by intrinsic tryptophan fluorescence and circular dichroism. Journal of Molecular Endocrinology 35: 211–223. 16216903 10.1677/jme.1.01830

[42] Torres NH, Ferreira LFR, Pinê Américo JH, Rossi de Moura Andrade GC, de Oliveira Freguglia RM, et al (2012) Analysis and occurrence of residues of the hormones estriol, 17α-ethinylestradiol and 17β-estradiol in urban water supply by HPLC-DAD. IOSR Journal of Engineering 2: 984–989.

[43] Mater N, Geret F, Castillo L, Faucet-Marquisa V, Albasia C, et al (2014) In vitro tests aiding ecological risk assessment of ciprofloxacin, tamoxifen and cyclophosphamide in range of concentrations released in hospital wastewater and surface water. Environment International 63: 191–200. 24317225 10.1016/j.envint.2013.11.011

[44] Gong J, Ran Y, Chen D, Yang Y, Zeng EY (2012) Association of endocrine-disrupting chemicals with total organic carbon in riverine water and suspended particulate matter from the Pearl River, China. Environmental Toxicology and Chemistry 31: 2456–2464. 22847724 10.1002/etc.1961

[45] Laws SC, Carey SA, Ferrell JM, Bodman GJ, Cooper RL (2000) Estrogenic activity of octylphenol, nonylphenol, bisphenol A and methoxychlor in rats. Toxicological Sciences 54: 154–167. 10746942 10.1093/toxsci/54.1.154

[46] Blair RM, Fang H, Branham WS, Hass BS, Dial SL, et al (2000) The estrogen receptor relative binding affinities of 188 natural and xenochemicals: structural diversity of ligands. Toxicological Sciences 54: 138–153. 10746941 10.1093/toxsci/54.1.138

[47] Aqai P, Gomez Blesa N, Major H, Pedotti M, Varani L, et al (2013) Receptor-based high-throughput screening and identification of estrogens in dietary supplements using bioaffinity liquid-chromatography ion mobility mass spectrometry. Analytical and Bioanalytical Chemistry 405: 9427–9436. 24081568 10.1007/s00216-013-7384-1

[48] Ricciardi C, Ferrante I, Castagna R, Frascella F, Marasso SL, et al (2013) Immunodetection of 17β-estradiol in serum at ppt level by microcantilever resonators. Biosensors and Bioelectronics 40: 407–411. 22964384 10.1016/j.bios.2012.08.043

[49] DeLano WL (2002) The PyMOL Molecular Graphics System.

[50] Zhang Y (2008) I-TASSER server for protein 3D structure prediction. BMC Bioinformatics 9: 1–8. 18215316 10.1186/1471-2105-9-40 PMC2245901

[51] Roy A, Kucukural A, Zhang Y (2010) I-TASSER: a unified platform for automated protein structure and function prediction. Nature Protocols 5: 725–738. 20360767 10.1038/nprot.2010.5 PMC2849174

[52] Pettersen EF, Goddard TD, Huang CC, Couch GS, Greenblatt DM, et al (2004) UCSF Chimera—a visualization system for exploratory research and analysis. Journal of Computational Chemistry 25: 1605–1612. 15264254 10.1002/jcc.20084

[53] O'Boyle NM, Banck M, James CA, Morley C, Vandermeersch T, et al (2011) Open Babel: An open chemical toolbox. Journal of Cheminformatics 3: 1–14. 21982300 10.1186/1758-2946-3-33 PMC3198950

[54] Grosdidier A, Zoete V, Michielin O (2011) Fast docking using the CHARMM force field with EADock DSS. Journal of Computational Chemistry 32: 2149–2159. 21541955 10.1002/jcc.21797

[55] Brooks BR, Brooks CLI, Mackerell ADJ, Nilsson L, Petrella RJ, et al (2009) CHARMM: the biomolecular simulation program. Journal of Computational Chemistry 30: 1545–1614. 19444816 10.1002/jcc.21287 PMC2810661

[56] Haberthur U, Caflisch A (2008) FACTS: fast analytical continuum treatment of solvation. Journal of Computational Chemistry 29: 701–715. 17918282 10.1002/jcc.20832

[57] Morris GM, Huey R, Lindstrom W, Sanner MF, Belew RK, et al.. (2009) Autodock4 and AutoDockTools4: automated docking with selective receptor flexiblity. 16. 10.1002/jcc.21256 PMC2760638 19399780

[58] Studier FW (1991) Use of bacteriophage T7 lysozyme to improve an inducible T7 expression system. Journal of molecular Biology 219: 37–44. 2023259 10.1016/0022-2836(91)90855-z

[59] Ahrens H, Schuh TJ, Rainish BL, Furlow JD, Gorski J, et al (1992) Overproduction of full-length and truncated human estrogen receptors in *Escherichia coli.* . Receptor 2: 77–92. 1472947

[60] Sreerama N, Woody RW (2000) Estimation of protein secondary structure from CD spectra: Comparison of CONTIN, SELCON and CDSSTR methods with an expanded reference set. Analitical Biochemistry 287: 252–260. 10.1006/abio.2000.4880 11112271

[61] Greenfield NJ (2006) Using circular dichroism collected as a function of temperature to determine the thermodynamics of protein unfolding and binding interactions. Nature Protocols 1: 2527–2535. 17406506 10.1038/nprot.2006.204 PMC2752288

